# The endoplasmic reticulum stress-related genes and molecular typing predicts prognosis and reveals characterization of tumor immune microenvironment in lung squamous cell carcinoma

**DOI:** 10.1007/s12672-024-00887-4

**Published:** 2024-02-16

**Authors:** Ruolan Wang, Yanhua Huang, Juan He, Shan Jin, Xin Li, Kun Tan, Wei Xia

**Affiliations:** 1 College of Pharmacy, Dali University, Dali, 671000 Yunnan China; 2 Department of Pharmacy, 920th Hospital of Joint Logistics Support Force of People’s Liberation Army, Kunming, 650032 Yunnan China; 3 Department of Procurement Management, 920th Hospital of Joint Logistics Support Force of People’s Liberation Army, Kunming, 650032 Yunnan China

**Keywords:** Endoplasmic reticulum stress, Lung squamous cell carcinoma (LUSC), Prognosis, Tumor immune microenvironment

## Abstract

**Background:**

Endoplasmic reticulum stress (ERS) acts critical roles on cell growth, proliferation, and metastasis in various cancers. However, the relationship between ERs and lung squamous cell carcinoma (LUSC) prognoses still remains unclear.

**Methods:**

The consensus clustering analysis of ERS-related genes and the differential expression analysis between clusters were investigated in LUSC based on TCGA database. Furthermore, ERS-related prognostic risk models were constructed by LASSO regression and Cox regression analyses. Then, the predictive effect of the risk model was evaluated by Kaplan–Meier, Cox regression, and ROC Curve analyses, as well as validated in the GEO cohort. According to the optimal threshold, patients with LUSC were divided into high- and low- risk groups, and somatic mutations, immune cell infiltration, chemotherapy response and immunotherapy effect were systematically analyzed.

**Results:**

Two ERS-related clusters were identified in patients with LUSC that had distinct patterns of immune cell infiltration. A 5-genes ERS-related prognostic risk model and nomogram were constructed and validated. Kaplan–Meier curves and Cox regression analysis showed that ERS risk score was an independent prognostic factor (p < 0.001, HR = 1.317, 95% CI = 1.159–1.496). Patients with low-risk scores presented significantly lower TIDE scores and significantly lower IC50 values for common chemotherapy drugs such as cisplatin and gemcitabine.

**Conclusion:**

ERS-related risk signature has certain prognostic value and may be a potential therapeutic target and prognostic biomarker for LUSC patients.

**Supplementary Information:**

The online version contains supplementary material available at 10.1007/s12672-024-00887-4.

## Introduction

Lung cancer is the common and most lethal malignancy worldwide [1]. According to the 2020 global cancer statistics, lung cancer ranks first in mortality and second in morbidity, respectively [2]. Except lung adenocarcinoma, lung squamous cell carcinoma (LUSC) is the most common histologic subtype of lung cancer, accounting for approximately 30% of all cases [3]. Despite great progress in the diagnosis and treatment of LUSC, the 5-year survival rate of patients with LUSC remains low [4]. Therefore, discovering promising diagnostic and prognostic biomarkers or drug targets is an urgent medical need.

The endoplasmic reticulum (ER) is the largest organelle in eukaryotic cells and is the main site of protein synthesis, processing and transportation [5]. ERS is a status imbalance of ER homeostasis caused by the accumulation of unfolded or misfolded proteins and changes of Ca + concentration [6]. As an important organelle, dysfunction of the ER has important implications for several cellular biological processes, such as lipid synthesis, protein folding, calcium storage, and signal transduction [7]. Furthermore, previous studies demonstrated that ERS was involved in the occurrence and malignant progression of various cancers such as lung cancer [8–10]. The continuous activation of ERS sensors enhances the tumorigenicity and metastasis potential of malignant cells, allowing them to acquire resistance through their mediated dormancy and immunosuppression, and ultimately survive chemotherapy [8, 11, 12]. However, ERS acts as an oncogenic factor only at moderate levels, while uncontrolled stress may also lead to cell death [13]. Intracellular reactive oxygen species (ROS) mediated ERS suppresses tumors in lung cancer cells [10]. Furthermore, the ERS pathway can be augmented by neutrophil arginase-1, which is discharged from activated apoptotic human neutrophils, and triggers apoptosis in cancer cells [14]. Therefore, it was reasonable to believe that ERS may be a valuable target for the treatment of malignant tumors. However, the detailed mechanism and clinical prognostic relationship of ERS in the occurrence and progression of LUSC are still unclear. Identification of certain potential biomarkers related to the occurrence and progression of LUSC would eventually contribute to better understanding of tumor development and further identification of candidate therapeutic targets.

In light of these backgrounds, we explored the patterns of ERS-related genes and the prognostic value of these genes in clinical practice based on TCGA dataset. We developed an independent prognostic ERS-related risk signature and validated it in the GEO cohort. Furthermore, this signature was used to predict tumor immune cell infiltration, chemotherapy response, and immunotherapy effect and discover potential small molecule drugs. This study might provide scientific foundation for exploring new prognostic biomarkers and treatments of LUSC.

## Materials and methods

### Data collection and processing

The Cancer Genome Atlas (TCGA) database (https://portal.gdc.cancer.gov/) was utilized to obtain RNA-sequencing data and clinical information. Additionally, the Gene Expression Omnibus (GEO) database (https://www.ncbi.nlm.nih.gov/geo/) was accessed to procure the GSE37745 dataset. The TCGA dataset comprised 49 normal lung tissues and 496 LUSC samples, from which gene expression profiles were extracted. The inclusion criteria were as follows: (1) samples were collected from patients with LUSC, and (2) corresponding clinical follow-up data were available. The exclusion criteria for the samples were as follows: (1) patients with no follow-up time, no survival status, and other clinical follow-up information missing; (2) patient samples with clinical data but no corresponding RNA-Seq data; (3) samples followed up for less than 30 days. After screening, this study included 469 LUSC samples along with corresponding clinical information (Additional file 2: Fig. S2). GSE37745 contained information of 66 LUSC samples.

### Consensus clustering analysis of ERS-related gene

Two ERS-related gene sets (GO RESPONSE TO ENDOPLASMIC RETICULUM STRESS and GO REGULATION OF RESPONSE TO ENDOPLASMIC RETICULUM STRESS) were downloaded from the Molecular Signature Database (MSigDB) v7.5 [15] (http://www.gsea-msigdb.org/gsea/msigdb/). A total of 260 ERS-related genes were obtained by merging and deduplication. Among them, 240 genes could be found in the TCGA dataset. Utilizing the “ConsensusClusterPlus” R package, we divided the TCGA dataset into two distinct clusters and analyzed the consensus cumulative distribution function (CDF) curves to determine the optimal number of clusters.

### Analysis of differential gene expression

The TCGA gene sequencing data were analyzed by using “Deseq2” [16] package, set thresholds as |log2FC|> 1.0 and Padj < 0.05 to identify differential genes in C1 and C2. Heatmaps were plotted by using the “pheatmap” package based on the results of differential gene expression analysis.

### Immunogenomic landscape analysis

The CIBERSORT algorithm was employed to assess variations in the infiltration levels of 22 immune cell types between two groups, namely C1 and C2, corresponding to high- and low-risk categories. The differential expression of eight potential inhibitory immune checkpoint genes was further analyzed, with statistical significance determined at p < 0.05. Tumor extent, stromal score, immune score, and estimated score of each tumor sample were calculated by the “estimate” package. The differences in immune cell infiltration between high-risk group and low-risk group were assessed using the single sample gene enrichment analysis (ssGSEA) algorithm.

### Analysis of drug sensitivity

The drug response of LUSC samples was analyzed using the “oncoPredict” [17] package to calculate the standardized half-maximal inhibitory concentration (IC50) of drug efficacy on the samples. A non-parametric test appropriate for this sample size, the Wilcox test, was used to examine all mean value comparisons. The correlation between model genes and drug sensitivity was analyzed by cellMiner database.

### Construction and validation of prognostic risk model related to ERS

Univariate cox analysis was used to assess the relationship between differentially expressed genes (DEGs) of C1 and C2 clusters and overall survival (OS) of LUSC patients. The TCGA dataset was randomly divided in a 1:1 ratio into two groups, namely the train set and the test set. Using Lasso regression and multivariate cox analysis, prognostic risk models associated with ERS were developed in the train group. The risk score for each sample was calculated as following formula: Risk score = Σ(Expi × Coef). In this formula, xi was the relative expression value of each selected gene and βi was the coefficient obtained from LASSO analysis. For validation, the same genes, regression coefficients and formula were applied in GEO dataset and test group to calculate the risk score. Subsequently, patients with LUSC were categorized into high- and low-risk groups based on the optimal threshold of risk score, and the OS was compared through K–M analysis. To validate the sensitivity and specificity of the prognostic model, ROC curves were computed. The distribution pattern of each risk group was evaluated through Principal Component Analysis (PCA) using the “stats” package. Nomogram were constructed by integrating clinical parameters and risk scores, and the predictive performance of nomogram, risk scores, and other clinical parameters was assessed by analyzing the ROC and the decision curve analysis (DCA) curves.

### Functional analysis

We conducted gene ontology (GO) and Kyoto Encyclopedia of Genes and Genomes (KEGG) pathway enrichment analyses, which included three key aspects that describe biological function: molecular function (MF), cellular component (CC), and biological process (BP). Padj < 0.05 for GO pathway and KEGG pathway were considered to be statistically significant. The relevant pathways and molecular mechanisms were assessed by downloading c2.cp.kegg.v7.5.1.symbols.gmt from the GSEA online web site and visualize the first five pathways, and Padj < 0.05 were considered to be statistically significant.

### Somatic mutation analysis

The “maf” data of somatic mutations in LUSC samples were obtained from the TCGA GDC database. Mutated genes were summarized and visualized using the “Maftools” R package.

### Statistical analysis

Statistical analysis was conducted using R Studio software (version 4.1.1). Survival distribution was estimated using K–M survival curve analysis. Prognostic value of stress-related characteristics of ERS was evaluated through Cox regression analysis, nomogram model, and time ROC curve analysis. All p-value < 0.05 were considered to be statistically significant.

## Results

### Consensus clustering of molecular subtypes based on ERS-related genes

**Fig. 1:**
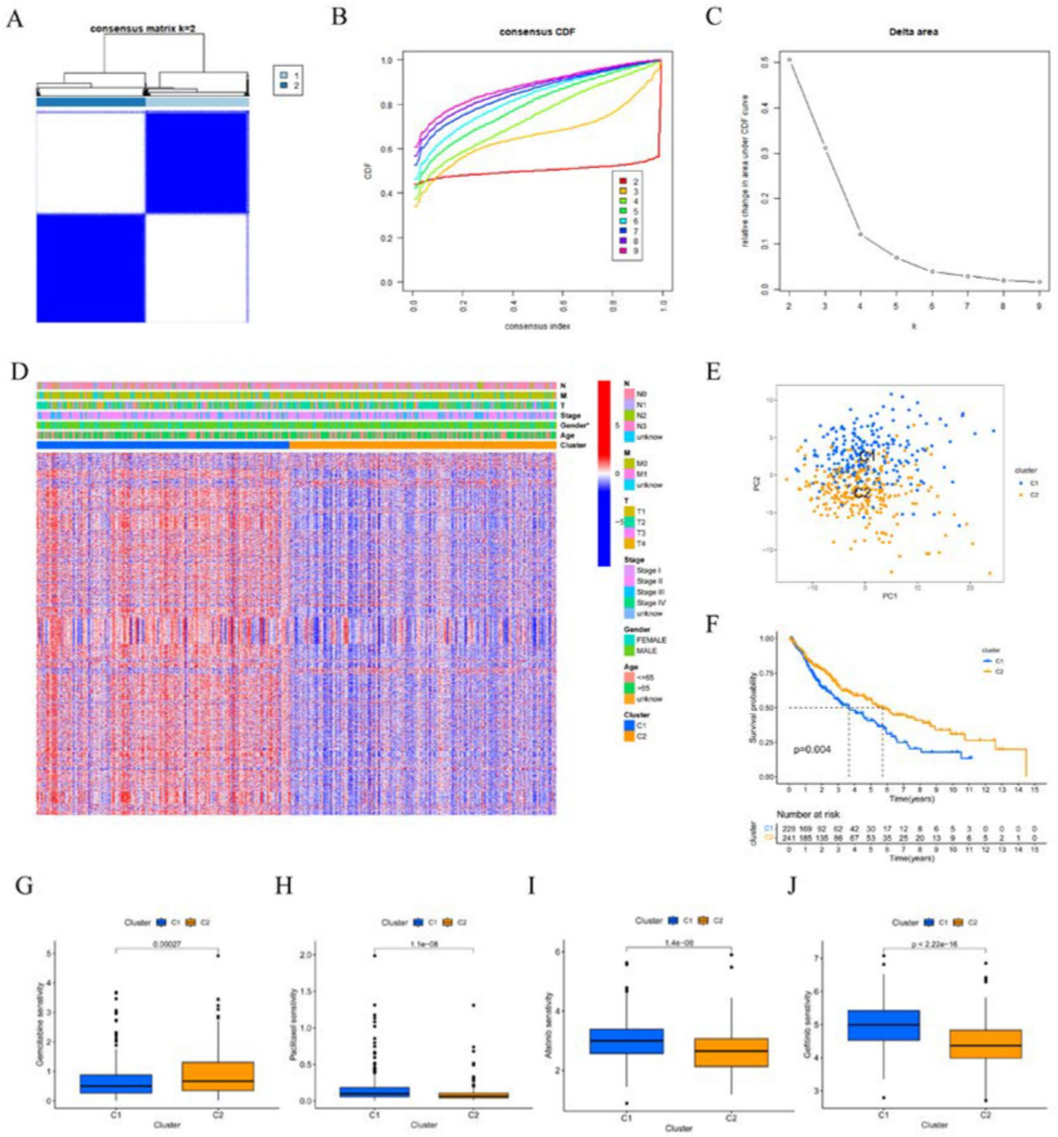
ERS-related clusters of LUSC. **A** Consensus cluster matrix of LUSC samples when *k* = 2. **B** CDF curves for *k* = 2-9. **C** Delta area for *k* = 2-9 in the consistent clustering model. **D** Heatmap of ERS-related genes and distribution of clinical parameters between two clusters. **E** PCA for two ERS-related clusters. **F** OS of two clusters. The IC50 values of (**G**) Gemcitabine, (**H**) Paclitaxel, (**I**) Afatinib, and (**J**) Gefitinib in two clusters. *p < 0.05

Consensus clustering based on ERS-related genes was conducted in this study. The clustering results and CDF curves revealed that the optimal clustering of LUSC samples from the TCGA dataset had two subtypes, namely C1 (n = 228) and C2 (n = 241) (Fig. 1A–C). Subsequently, differential analysis for the two subtypes was performed followed by the examination of the correlation between ERS-related typing and clinical parameters as well as the expression patterns of genes associated with ERS, which is exhibited in the heatmap (Fig. 1D). Furthermore, PCA plots were analyzed to show that the two subtypes were distributed in different sections (Fig. 1E). K–M analysis demonstrated that LUSC samples in subtype C2 exhibited better survival outcomes than those in subtype C1 (Fig. 1F).

### Evaluation of drug response of the clusters

Gemcitabine, Paclitaxel, Afatinib, and Gefitinib are commonly used in the clinical treatment of LUSC. The response of these four commonly used drugs was assessed by analyzing the IC50 values. The results showed that patients with C1 cluster had lower IC50 values for Gemcitabine (Fig. 1G); while patients with C2 had lower IC50 values for Paclitaxel, Afatinib and Gefitinib (Fig. 1H–J).

### Identification of immune cell infiltration for the two subtypes

**Fig. 2:**
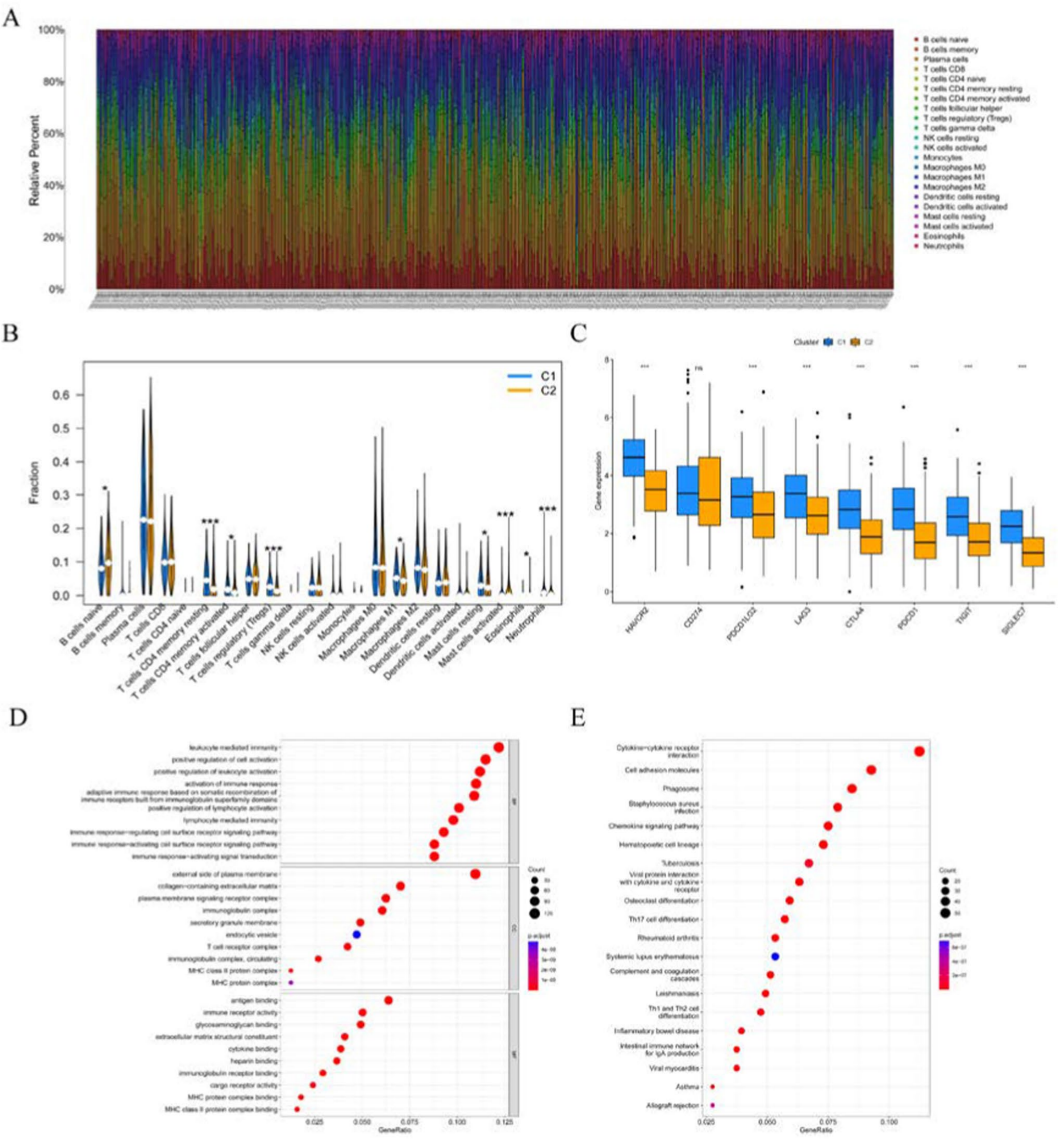
Levels of immune cell infiltration and between the two clusters. **A, B** Abundance of the 22 immune cell types belonging to the two clusters. **C** Differential expression of the inhibitory immune checkpoints between two clusters. **D, E** GO and KEGG pathways analysis of the DEGs between the two clusters. *p < 0.05, **p < 0.01, ***p < 0.001

The CIBERSORT algorithm was used to study the infiltration level of 22 immune cell types. The results showed that the infiltration level of native B cells, activated mast cells and eosinophils was higher in the C2 subtype than the corresponding infiltration levels in the C1 subtype (Fig. 2A, B). In contrast, it showed lower infiltration levels of resting memory CD4 T cells, activated memory CD4 T cells, regulatory T cells (Tregs), macrophages M1, mast cells resting, and neutrophils in C2 cluster than in C1 cluster. The expression levels of 8 inhibitory immune checkpoints were examined to better analyze the tumor immune microenvironment (TIME) of both subtypes. Except for the expression level of CD274, which did not differ, the expression of the other seven suppressive immune checkpoints were found to be significantly up-regulated in C1 than that in C2 (Fig. 2C).

### Functional enrichment analysis of two clusters DEGs

GO and KEGG analyses were used to investigate the potential biological functions and pathways of DEGs. These DEGs were enriched in immune-related biological processes and molecular functions, such as leukocyte mediated immunity, positive regulation of leukocyte activation, activation of immune responses, immunoglobulin complex, and immune receptor activity (Fig. 2D). KEGG analyses results also showed enrichment of immune-related pathways, include cytokine-cytokine receptor interaction, cell adhesion molecules, phagosome, and chemokine signaling pathway (Fig. 2E).

### Construction and validation of risk models

**Fig. 3:**
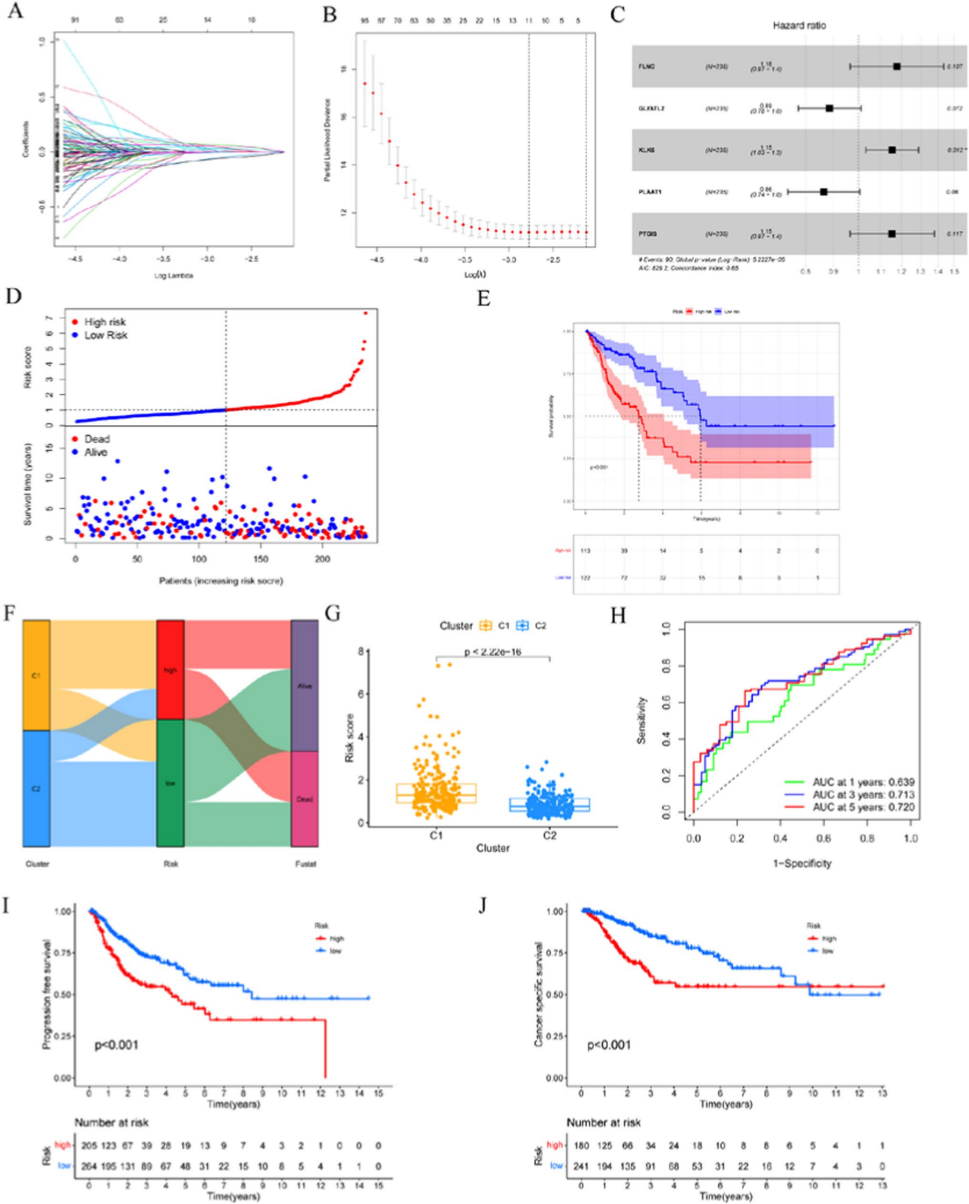
Construction of the prognostic risk model. **A, B** LASSO regression analysis was used to calculate the optimal lambda. Min value = 11. **C** Forest maps of five genes obtained by multivariate Cox analysis. **D** Survival state and **E** Kaplan–Meier survival curve of the TCGA-training set. **F** Alluvial diagram of cluster distributions with distinct risk score and clinical outcomes. **G** Difference of risk score in the two clusters. **H** ROC curve of the TCGA-training set. **I, J** Progression free survival and cancer-specific survival of the TCGA set

**Fig. 4:**
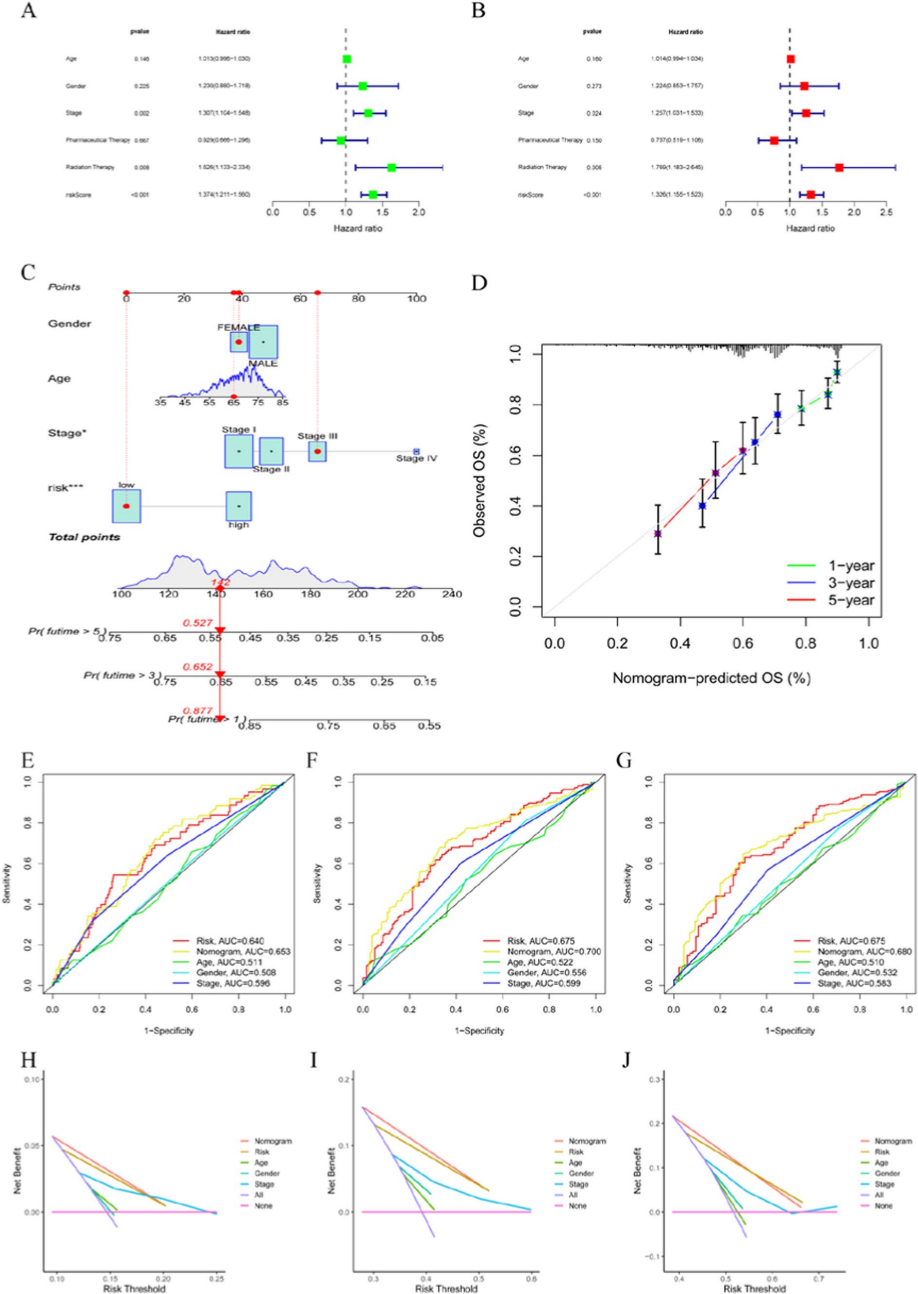
Construction and validation of a nomogram for predicting OS in TCGA dataset. **A, B** Univariate and multivariate regression analysis. **C** Construction of a nomogram based on age, gender, stage and risk score. **D** Verification of the predictive accuracy of the nomogram by calibration curves. **E–G** ROC curves of the nomogram, risk score, and clinical parameters for predicting the 1-, 3-and 5-year OS. **H–J** DCA curves for comparing the net survival benefit of the nomogram, risk score, and clinical parameters

To further investigate the prognostic features between distinct clusters, we performed Lasso-Cox regression analysis on 1243 DEGs between the two subtypes and constructed a prognostic risk model related to ERS (Fig. 3A–C). The expression of five genes used to construct the prognostic risk model was shown in the heatmap (Additional file 1: Fig. S1A). The risk score was calculated by the following formula: 0.1628*Expression*FLNC* − 0.1216*Expression*GLYATL2* + 0.1432*Expression*KLK6*—0.1462*Expression*PLAAT1* + 0.1427*Expression*PTGIS*. LUSC patients were divided into two risk groups according to the optimal threshold of risk score (Fig. 3D). Results from PCA and t-SNE indicated that LUSC samples belonging to different risk groups were distributed in distinct regions (Additional file 1: Fig. S1B, C). Correlation analysis of risk scores and survival status showed that higher risk score was associated with higher mortality (Fig. 3D). Patients in the high-risk group had a worse prognosis than those in the low-risk group (p < 0.001) (Fig. 3E). The Sankey diagram showed that most C1 samples were in the high-risk group and had a poor prognosis, validating the results of the previous survival analysis (Fig. 3F, G). The areas under ROC curves for 1, 3 and 5 years were 0.639, 0.713 and 0.720 (Fig. 3H), respectively, which indicates that the model has good prediction efficiency. The application of the risk model showed significant differences in progression free survival and cancer-specific survival (p < 0.001) (Fig. 3I, J). To verify the predictive value of this risk model, we used the test set and GSE37745 to verify that the high-risk group had significantly higher mortality than the low-risk group (p < 0.05) (Additional file 1: Fig. S1G, M). Similarly, PCA and t-SNE distributed the samples of the two risk groups in different regions (Additional file 1: Fig. S1E, F, K, L). The AUC values for 1, 3, and 5 years were relatively high in both the test set (0.652, 0.652, and 0.659) and GSE37745 (0.647, 0.625, and 0.608), highlighting the stability of this risk model (Additional file 1: Fig. S1I, O). Univariate and multivariate regression analyses showed that risk score was an independent risk factor (p < 0.001, HR = 1.317, 95%CI = 1.159–1.496) (Fig. 4A, B). According to the results of multivariate regression analyses, a nomogram was constructed based on clinical parameters and risk scores (Fig. 4C). Calibration curves showed that the predicted OS was consistent with the actual observed OS (Fig. 4D). Multi-indicator ROC curves combining clinical factors, risk scores, and nomograms showed that AUC values on 1, 3, and 5-year nomograms were higher than other parameters (Fig. 4E–G). DCA analysis showed that the use of the nomogram was superior to other clinical factors in predicting patient survival (Fig. 4H–J).

### Association of the risk signature with GSEA and somatic mutations in LUSC

**Fig. 5:**
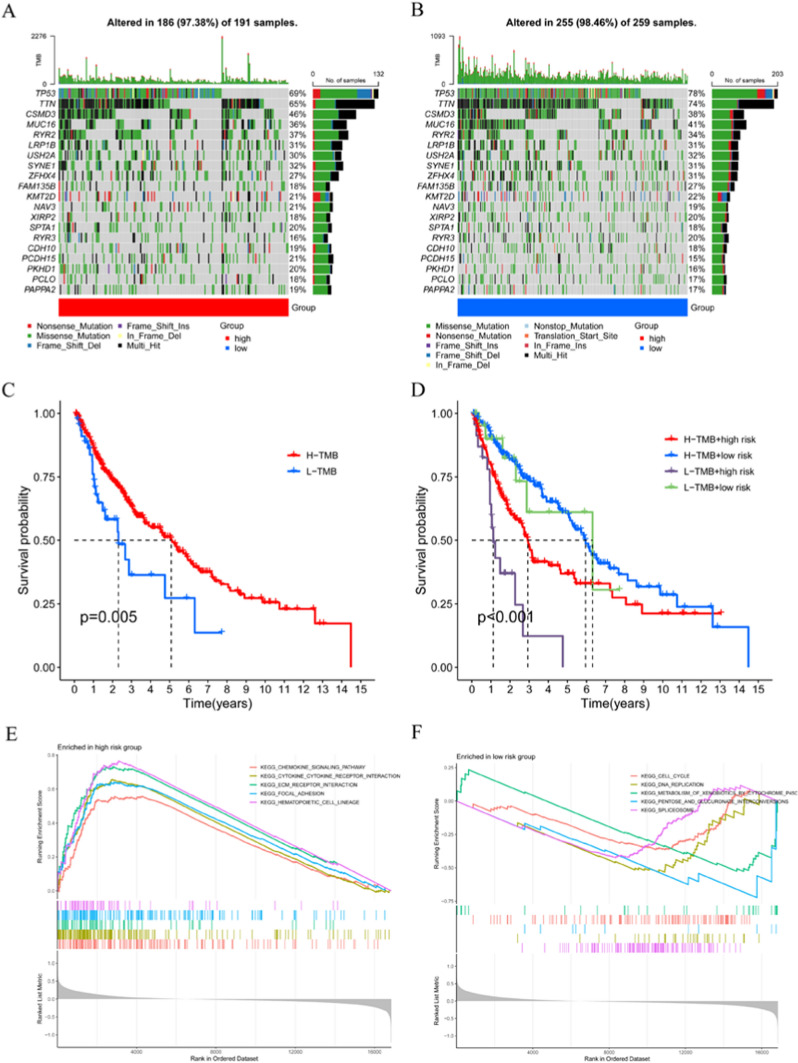
The somatic mutations and functional analysis at different risk score. **A, B** Distinct somatic mutations identified by comparing LUSC with high- and low-risk score. **C** Kaplan-Meier OS curves for different TMB. **D** Kaplan-Meier OS curves for different TMB and risk score subgroups. **E, F** GSEA analysis in TCGA dataset

We noted different somatic mutation profiles in the high- and low- risk groups (Fig. 5A, B). Although TP53, TTN, CSMD3 and MUC16 were the most common mutations, the relative frequencies differed between the two groups. The low-risk group showed a high frequency of TP53 and TTN mutations, accounting for 78% and 74% of the total; while the high-risk group was only 69% and 65%. Moreover, the survival rates were significantly lower in the low-tumor mutation burden (TMB) group compared to the high-TMB group (Fig. 5C). Combined analysis of TMB and risk scores showed that the high-TMB and low-risk group had a better prognosis than that of the other three groups (Fig. 5D).

GSEA results showed that the high-risk group was mainly associated with “chemokine signaling pathway”, “cytokine-cytokine receptor interaction”, “ecm receptor interaction”, “focal adhesion” and “hematopoietic cell lineage” (Fig. 5E). The low-risk group was mainly associated with “cell cycle”, “DNA replication”, “metabolism of xenobiotics by cytochrome”, “pentose and glucuronate interconversion” and “spliceosome” (Fig. 5F). These results suggested that the low-risk group was mainly involved in cell cycle and metabolism-related signaling pathways; while the high-risk group was mainly involved in immune and cell transfer-related signaling pathways.

### Association of the risk signature with tumor microenvironment

**Fig. 6:**
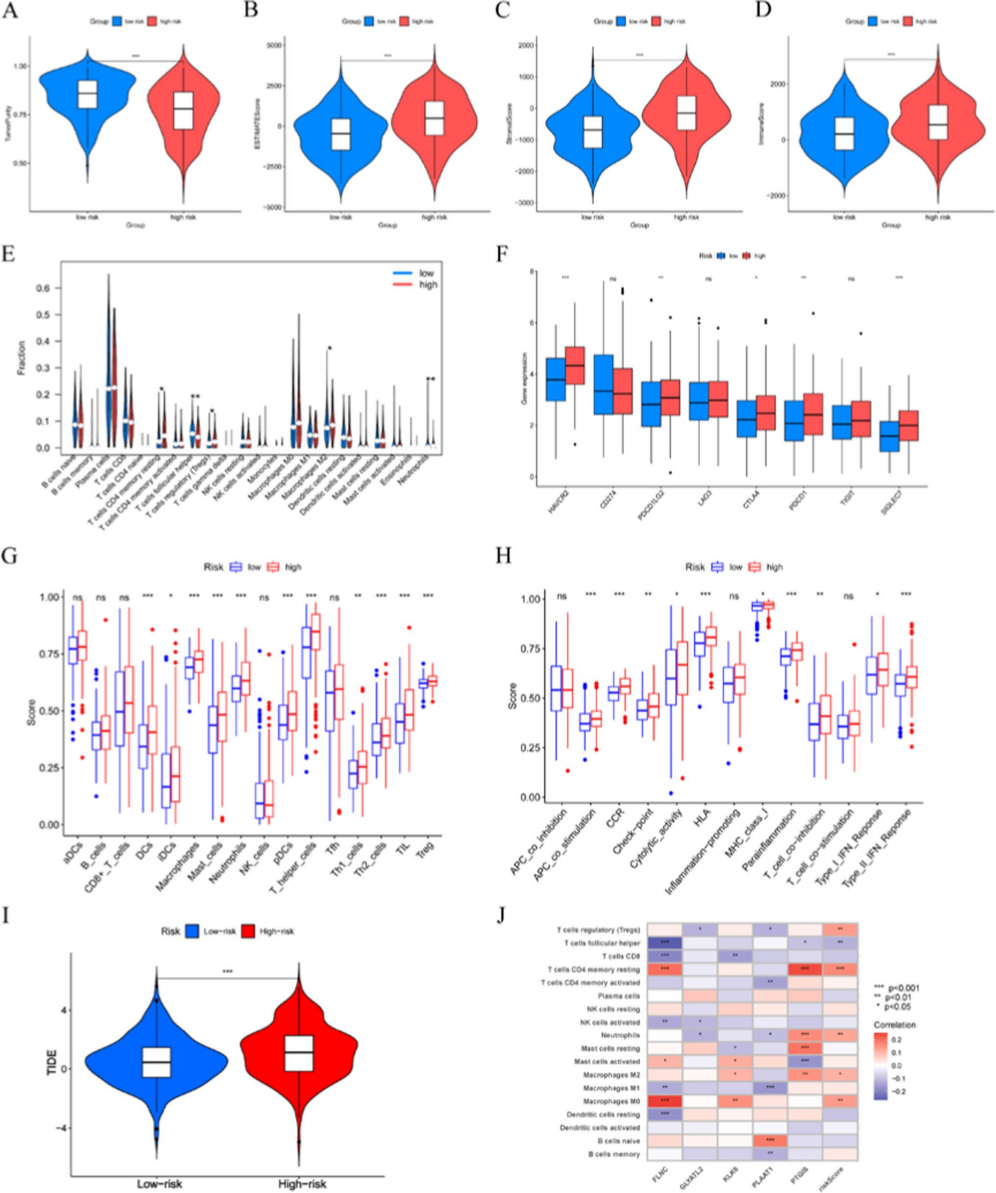
The immune microenvironment of LUSC tissues at different risk score. **A** Tumor purity, **B** ESTIMATE score, **C** stromal score and (**D**) immune score between high- and low-risk populations. **E** Immune cell component between high- and low-risk groups. **F** Differential expression of the inhibitory immune checkpoints between two clusters. **G, H** 29 types of immune signatures between high- and low-risk groups. **I** TIDE scores were used to compare immunotherapy efficacy between high- and low-risk groups. **J** Correlations between the abundance of immune cells and five genes in the proposed signature

We analyzed the immune microenvironment between the high- and low-risk groups. The ESTIMATE algorithm revealed that the high-risk group had lower tumor purity and higher estimate score, stromal score and immune score (Fig. 6A–D). The CIBERSORT algorithm revealed a statistically significant decrease in the levels of T cells follicular helper in the high-risk group compared to the low-risk group (Fig. 6E, F). Furthermore, the immunosuppressive Macrophages M2 and Tregs levels were markedly elevated in the high-risk group, indicating a higher immunosuppressive activity that could potentially facilitate tumor progression. The ssGSEA algorithm demonstrated a higher immune cell infiltration and more immune-related functions and pathways in the high-risk group compared to the low-risk group (Fig. 6G, H). These findings suggested that the high-risk group might have a relatively stronger immune response but concurrently with increased immunosuppressive activity. Higher Tumor Immune Dysfunction and Exclusion (TIDE) scores were associated with shorter survival (Fig. 6I). Higher TIDE scores in the high-risk group suggested a poorer response to immune checkpoint inhibitors; in other words, patients in the low-risk group could benefit more from immunotherapy. In addition, the five genes signature used to construct the model were significantly associated with multiple immune cell infiltrations (Fig. 6J).

### Prediction of chemotherapy effects by the risk signature

**Fig. 7:**
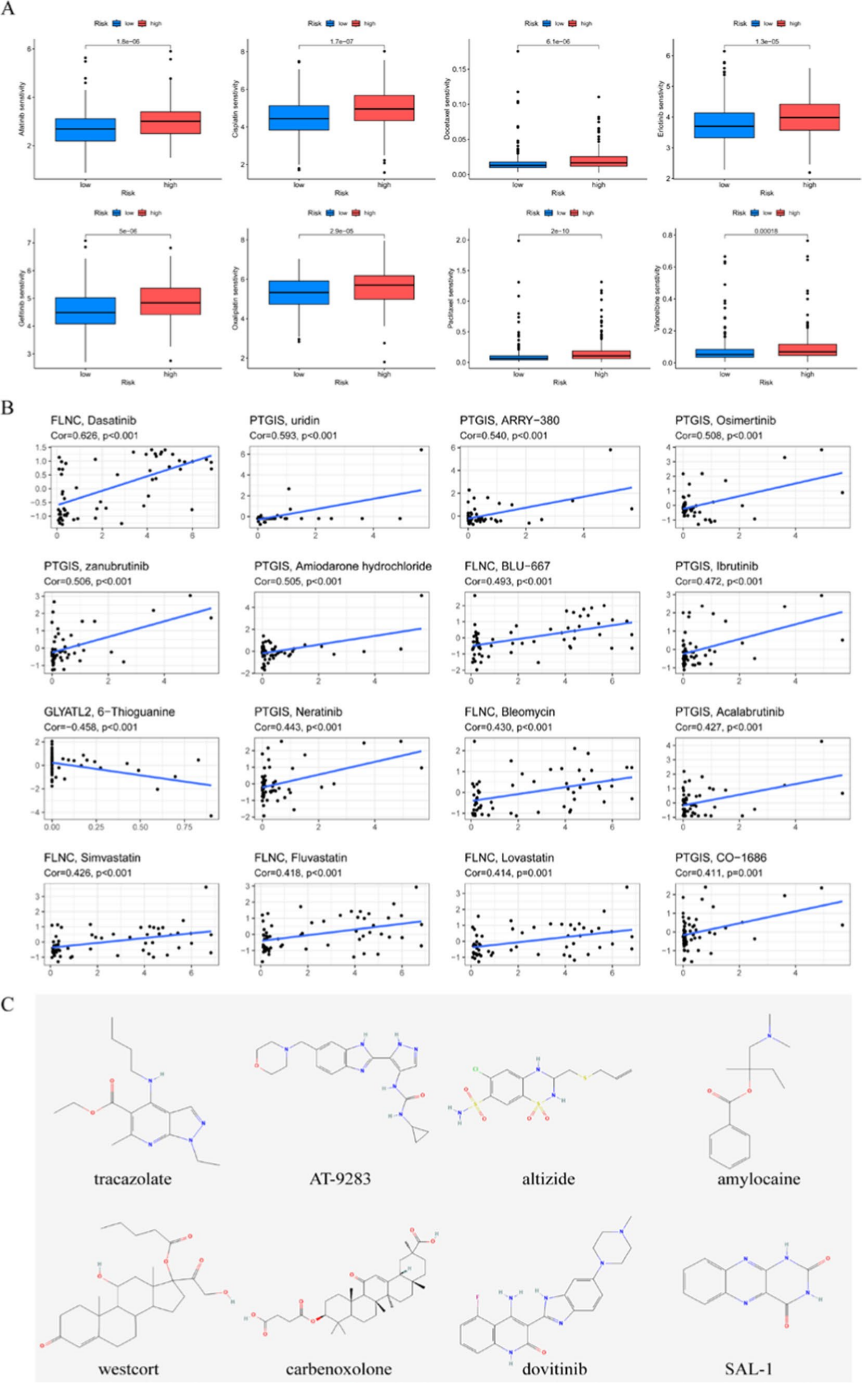
Effect of ERS-related risk signature on drug sensitivity. **A** Differences in response to commonly used chemotherapy drugs between high- and low-risk groups. **B** Construct the correlation between signature gene and drug sensitivity. **C** The 2D chemical structures of eight small molecule drugs

First, data on the response of patients in high- and low-risk to common chemotherapeutic drugs were downloaded from the GDSC database. The results showed that many common chemotherapeutic drugs for LUSC differed significantly between the two groups (Fig. 7A). Gemcitabine and Cisplatin, as the most common chemotherapy medicines for LUSC, exhibited significantly lower IC50 values in the low-risk group than in the high-risk group (p < 0.05), indicating that Gemcitabine and Cisplatin might be more effective in the low-risk group. Nevertheless, the effect of these drugs in the treatment of LUSC patients remains to be further proven in future clinical studies. The relationship between model genes and drug sensitivity was analyzed from the cellMiner database, and the scatterplot of the top 16 drugs with the highest correlation between genes and drug sensitivity was drawn (Fig. 7B). A positive correlation indicates that higher gene expression is more sensitive to drugs, while a negative correlation indicates that higher gene expression is more resistant to drugs. Among them, the up-regulation of FLNC and PTGIS expression may lead to enhanced sensitivity of patients to most drugs. These findings imply that changes in the expression of genes constructing ERS-related signature may be useful in predicting medication response and serving as prospective therapeutic targets.

### Prediction of potential drugs

We analyzed DEGs between the high- and low-risk groups to screen for novel therapeutic compounds for the treatment of LUSC. Among the 99 DEGs studied, there were 64 up-regulated genes and 35 down-regulated genes. As shown in Table 1, the top 8 potential novel therapeutic small molecule compounds with the highest negative scores were screened (tracazolate, AT-9283, altizide, amylocaine, westcort, carbenoxolone, dovitinib and SAL-1) and their corresponding mechanisms of action (MoA) were explored as well. Among them, AT-9283 acts through 7 kinds of MoAs. Dovitinib acts through epidermal growth factor receptor (EGFR) inhibitors, fibroblast growth factor receptor (FGFR) inhibitors, platelet-derived growth factor receptor (PDGFR) inhibitors, and vascular endothelial growth factor receptor (VEGFR) inhibitors. The 2D chemical structures of each small molecule compound were also downloaded and are shown in Fig. 7C.

**Table 1:** 8 compounds with the highest negative enrichment score from Cmap database

Rank	Score	Compound	MOA
1	− 91.18	tracazolate	GABA receptor modulator
2	− 91.01	AT-9283	JAK inhibitor, Aurora kinase inhibitor, et al.
3	− 79.97	altizide	Thiazide diuretic
4	− 76.5	amylocaine	Local anesthetic
5	− 76.22	westcort	Glucocorticoid receptor agonist
6	− 73.58	carbenoxolone	11-beta-HSD1 inhibitor
7	− 73.21	dovitinib	EGFR inhibitor, FLT3 inhibitor, et al.
8	− 70.44	SAL-1	Adenosine receptor antagonist

## Discussion

Under the conditions of protein homeostasis in normal cells, sensors of ERS, including ATF6, IRE1a and PERK are inactivated, whereas in the tumor microenvironment (TME), multiple factors such as hypoxia [18], abnormal nutrient supply [19] and ROS accumulation [20] interfere with protein folding in the ER, thereby driving ERS in cancer cells. It was found that ERS was closely associated with the development of lung cancer [10]. For example, previous study showed that ERS promoted the process of epithelial-mesenchymal transition and cell invasion in LUAD [21]. Shu et al. found that ERS was associated with the efficacy of targeted therapy and immunotherapy for LUAD [22]. In addition, cancer cells could acquire resistance and survive chemotherapy through ERS-mediated dormancy and immunosuppression [8, 11, 12]. Further studies found that ERS was associated with cisplatin resistance in lung cells [23]. However, no studies have investigated the relationship between ERS-related genes and LUSC prognosis. Thus, we speculate that ERS has been largely overlooked as a novel prognostic marker for LUSC. In this work, we present several new findings: To our knowledge, this is the first time that an ERS-derived prognostic model has been developed for evaluating the prognosis of patients with LUSC. Second, we validated this model using the independent GSE37745 cohort. Importantly, we assessed the responses of different clusters or risk populations to commonly used medications for LUSC and predicted several potential small-molecule compounds.

We incorporated a comprehensive panel of ERS genes into our analysis of LUSC, which aims to enhance our comprehension of the involvement of ERS in the TME of LUSC and potentially facilitate the advancement of novel therapeutic approaches. The ERS-related risk model showed strong predictive effects in both the independent test set and the external validation set, which indicated that our risk model was reliable and effective. The result of the correlation between the risk score and the TIME showed that the immune scores of patients in the high-risk group were significantly higher than that in the low-risk group, and the abundance of tumor immune cells infiltration was also significantly different between the two groups. Based on our findings, the collective expression pattern of ERS-related genes appears to play a critical role in facilitating cancer cell adaptation and growth, ultimately resulting in an unfavorable prognosis for patients. Thus, ERS as a reliable predictor of LUSC prognosis and response to immunotherapy might provide valuable insights into the establishment of effective LUSC therapies.

Complementary studies targeting ERS and modulating the immunosuppressive TME component will become promising solid tumor therapies that target ERS-based LUSC. We found that C1 subtype samples had poorer survival than C2, suggesting that ERS-related gene patterns might be involved in tumor progression. In this work, we assessed the percentage of various cells in the TIME using CIBERSORT and ESTIMATE revealing that the C1 cluster population presented an immunosuppressive microenvironment landscape, which could be one of the reasons for treatment resistance in these patients. Moreover, the two subtypes had different levels of immune cell infiltration, which might be related to the significantly upregulated levels of Tregs and neutrophil infiltration observed in C1. Tregs, which are potent immunosuppressive cells in the immune system, can promote cancer progression by suppressing antitumor immunity [24]. They were heavily infiltrated in various tumors and often associated with poor clinical outcomes [25]. Neutrophils, on the other hand, are known to be involved in all stages of carcinogenesis [26]. High levels of neutrophil infiltration were related with poor survival in a range of malignancies, including lung cancer [27, 28]. In addition, the function of differential genes between the two clusters was enriched in immune-related and other functions. Furthermore, we analyzed the inhibitory immune checkpoint genes such as programmed cell death protein 1 (PD-1), cytotoxic T lymphocyte protein 4 (CTLA4), lymphocyte activation gene 3 protein (LAG3), and T cell immunoreceptor with immunoglobulin and ITIM domain (TIGIT), which participate in T cell dysfunction and malfunction of anti-tumor immunity[29], and discovered that they were significantly up-regulated in the C1 cluster. In the two subtypes mentioned above, CD8 + T cell presence was not statistically significant. Despite the fact that CD8 + T cell usually regarded as a positive regulation of anti-tumor immunity and signs of a good prognosis, and considering the TME as a complicated and elaborate regulation network, we speculate that the overall effect of ER stress is closely correlated with T cell exhaustion and formation of an immunosuppressive TME in LUSC. This is consistent with previous reports that PERK pathway is involved in the immunosuppressive function of MDSC [30]. These results provide further evidence that ERS might indeed play a pivotal role in the regulation of TME. A high expression level of inhibitory immune checkpoint genes hampers the immune response and facilitates the immune escape process in cancers. Presently, immune checkpoint inhibitors targeting CTLA4, LAG3, PD-1, and PD-1 ligand 1 (PD-L1) have been approved for clinical treatment, and some progress has been achieved in this field [31–33]. Together, these data are important for optimizing personalized, targeted treatment strategies for different sub-clusters.

Among these model genes (*FLNC*, *KLK6*, *PTGIS*, *PLAAT1* and *GLYATL2*), *FLNC* is an actin-binding protein that regulates the actin cytoskeleton in cells and is involved in cancer metastasis. As for the exact effect of *FLNC* on the prognosis of cancers, previous studies reported inconsistent results. Over-expression of *FLNC* in gastric and prostate cancers inhibited the proliferation and metastasis of tumor cells, and it was associated with a good prognosis [34]. However, several studies indicated that *FLNC* might promote tumor progression, migration and invasion [35, 36]. For instance, it was demonstrated that *FLNC* enhances the aggressiveness of glioblastoma cells by inducing MMP2 activation, leading to poor prognosis [35]. High expression of *FLNC* promoted lymphatic invasion and metastasis of esophageal squamous cell carcinoma by regulating Rho GTPase [36]. Therefore, this study system analyzed the role of *FLNC* expression in LUSC patients. The result suggested a significant association between high *FLNC* expression and poorer OS in LUSC, which indicated that overexpression of *FLNC* might contribute to poor prognosis.

*KLK6* is a secreted serine protease, which promotes the production of TNF-α by macrophages through PAR1 in the TME to stimulate the production of CXCL1 in tumor cells, thereby promoting tumor growth and metastasis [37]. In non-small cell lung cancer, high expression of *KLK6* was an indicator of tumor proliferation and poor prognosis [38]; *KLK6* was possible to be a potential novel diagnostic biomarker for LUSC [39]. These previous studies supported our results that high expression of *KLK6* was associated with poor prognosis in patients with LUSC.

*PTGIS* and *PLAAT1* might serve as new therapeutic targets for LUSC as well as biomarkers for prognosis and tumor immunity [40]. *PLAAT1* (*HRASLS*) regulated cell proliferation, tumor suppression, and phospholipid metabolism*.* [41]. Furthermore, studies have demonstrated its ability to promote the degradation of lens organelles such as the ER and lysosomes [42]. *PTGIS* is a member of the cytochrome P450 family and it is a key gene for synthesis of PGI2 and thus could indirectly affect normal inflammatory responses activation and differentiation of immune cell by catalyzing PGI2 [43, 44]. Dai et al. found that high expression of *PTGIS* promoted infiltration of TAMs and Tregs in the TME and worsened the prognosis of patients with lung, ovarian and gastric cancers [45]. In ovarian cancer, it was driven by PGI2/PTGIR to induce pro-tumor and immunosuppression [46]. Furthermore, Lei et al. discovered in his experimental study that contrary to *PLAAT1*'s inhibition of proliferation, migration and invasion of LUSC, *PTGIS* promoted that [40]. This was consistent with our study and further demonstrated the reliability of the risk model we constructed.

*GLYATL2* is a member of the glycine-binding enzyme gene family that localizes to the ER and belongs to the class of ER-associated proteins. The N-acyl glycine produced was anti-inflammatory and anti-proliferative, and its high level of expression in the skin and lung might indicated an important role in barrier function and immune response [47]. Given this, we speculated that the observed association between elevated *GLYATL2* expression and good prognosis in LUSC might be due to its biological functions. Whereas four of the five model genes were involved in the progression of multiple cancers, including LUSC, and were not only significantly associated with patient survival and prognosis, but their expression patterns were also associated with tumorigenesis and regulation of the TIME, which indicated that the results of our bioinformatics analysis were meaningful to a certain extent. Therefore, the ERS risk model might well predict the prognosis of LUSC and the immunotherapy efficacy.

Assessing the comprehensive characterization of immune-related genes and pathways helps to understand the association between cancer stemness and TME in lung cancer [48]. Functional analysis showed that high-risk populations were mainly involved in immune-related functions and signaling pathways, suggesting an interaction between ERS and LUSC immune response. Chemokines were found to be involved in promoting tumor heterogeneity by maintaining or promoting a stem cell-like phenotype. CXCR4/CXCL12 interactions contributed to the promotion of tumor-initiating cells in lung cancer, which was associated with chemotherapy resistance [49]. It has been shown that ERS could play an important role in tumor development through its immunomodulatory function [10]. Accordingly, we found that although patients in the high-risk group had a high immune infiltration profile, they also had a large number of immunosuppressive cells, such as Treg cells and M2 subtype macrophages. This was consistent with the immunosuppressive role of ERS in cancer reported by Cubillos-Ruiz et al. [8]. Compared to the low-risk group, the high expression of suppressive immune checkpoints and the enrichment of tumor immunosuppressive cells in the high-risk group suggest that the model successfully differentiates the immune type of LUSC. This suggested that ERS could regulate the immune microenvironment of LUSC and thus affect the prognosis of LUSC patients. Meanwhile, TIDE analysis showed that the TIDE score in the high-risk group was significantly higher than that in the low-risk group, indicating that ICI treatment was less effective in high-risk patients. Notably, the higher frequency of mutations in the low-risk group also suggests that they may be better able to benefit from immunotherapy [50]. In addition, we further analyzed the differences in the efficacy of commonly used chemotherapy drugs in the high- and low- risk groups and found that patients in the low-risk group were more sensitive to common chemotherapy drugs such as cisplatin. These results suggested that ERS characteristics might also be a reasonable and effective method for screening patients receiving chemotherapy. Thus, these findings suggested that the risk model we constructed could be used as an effective indicator to assess the response of LUSC patients to chemotherapy and immunotherapy and might provide useful advice for the future treatment of these patients.

Nevertheless, our study existed some limitations that should be acknowledged. Firstly, the retrospective design of our investigation called for further validation of the prognostic risk model through prospective clinical studies involving larger sample sizes. Secondly, our analysis, and hence conclusions, are based on data obtained from public databases, which may accordingly have led to inherent case selection bias. In addition, although we extensively discussed the prognostic value and biological implications of ERS in LUSC, their specific molecular mechanisms of action in LUSC still need to be verified by in vivo and in vitro experiments. Finally, we predicted several latent compounds for targeting LUSC patients, whose toxicological responses should also be considered in future studies.

## Conclusion

We categorized LUSC samples into two clusters based on their association with ERS and varying levels of prognosis and immune infiltration, and constructed risk models with good accuracy based on the DEGs between the two clusters. Our analysis revealed distinct immune characteristics between the high-risk and low-risk groups. Therefore, our findings might provide an important reference for clinicians in clinical diagnosis, prognostic analysis and achieving individualized comprehensive treatment of LUSC.

### Supplementary Information

**Additional file 1: Fig. S1** Validation of prognostic signature for LUSC.

**Additional file 2: Fig. S2** LUSC patients screening process.

## Data Availability

The relevant data of LUSC patients used in this study were obtained from TCGA and GEO databases.

## Abbreviations

AUC: Area under the curve
ATF6: Activating transcription factor 6
CDF: Cumulative distribution function
CMap: ConnectivityMap
DCA: Decision curve analysis
DEGs: Differentially expressed genes
ER: Endoplasmic reticulum
FDR: False discovery rate
FLNC: Filamin C
GDSC: Genomics of drug sensitivity in cancer
GEO: Gene expression omnibus
GLYATL2: Glycine-N-acyltransferase-like 2
GO: Gene ontology
GSEA: Gene set enrichment analysis
IC50: 50% Inhibitory concentration
ICIs: Immune checkpoint inhibitors
IRE1a: Inositol-requiring enzyme-1α
KEGG: Kyoto encyclopedia of genes and genomes
KLK6: Kallikrein-related peptidase 6
LASSO: Least absolute shrinkage and selection operator
LUAD: Lung adenocarcinoma
LUSC: Lung squamous cell carcinoma
MOA: Mechanisms of action
NSCLC: Non-small cell lung cancer
OS: Overall survival
PCA: Principal component analysis
PERK: Protein kinase R-like endoplasmic reticulum kinase
PTGIS: Prostaglandin I2 (prostacyclin) synthase
ROC: Receiver operating characteristic
ssGSEA: Single-sample gene set enrichment analysis
TCGA: The Cancer Genome Atlas
TIDE: Tumor Immune Dysfunction and Exclusion
TMB: Tumor mutational burden
TNF: Tumor necrosis factor
TP53: Tumor Protein P53
Tregs: T cells regulatory
t-SNE: T-distributed stochastic neighbor embedding
TTN: Titin
PD-1: Programmed cell death protein 1
CTLA4: Cytotoxic T lymphocyte protein 4
LAG3: Lymphocyte activation gene 3 protein
TIGIT: T cell immunoreceptor with immunoglobulin and ITIM domain
PD-L1: Programmed cell death protein 1 ligand 1
