# Supplementary figures and images for: The endoplasmic reticulum stress-related genes and molecular typing predicts prognosis and reveals characterization of tumor immune microenvironment in lung squamous cell carcinoma
Source: Discov Oncol. 2024 Feb 16;15:37. doi: 10.1007/s12672-024-00887-4 (PMC10873263; doi:10.1007/s12672-024-00887-4)

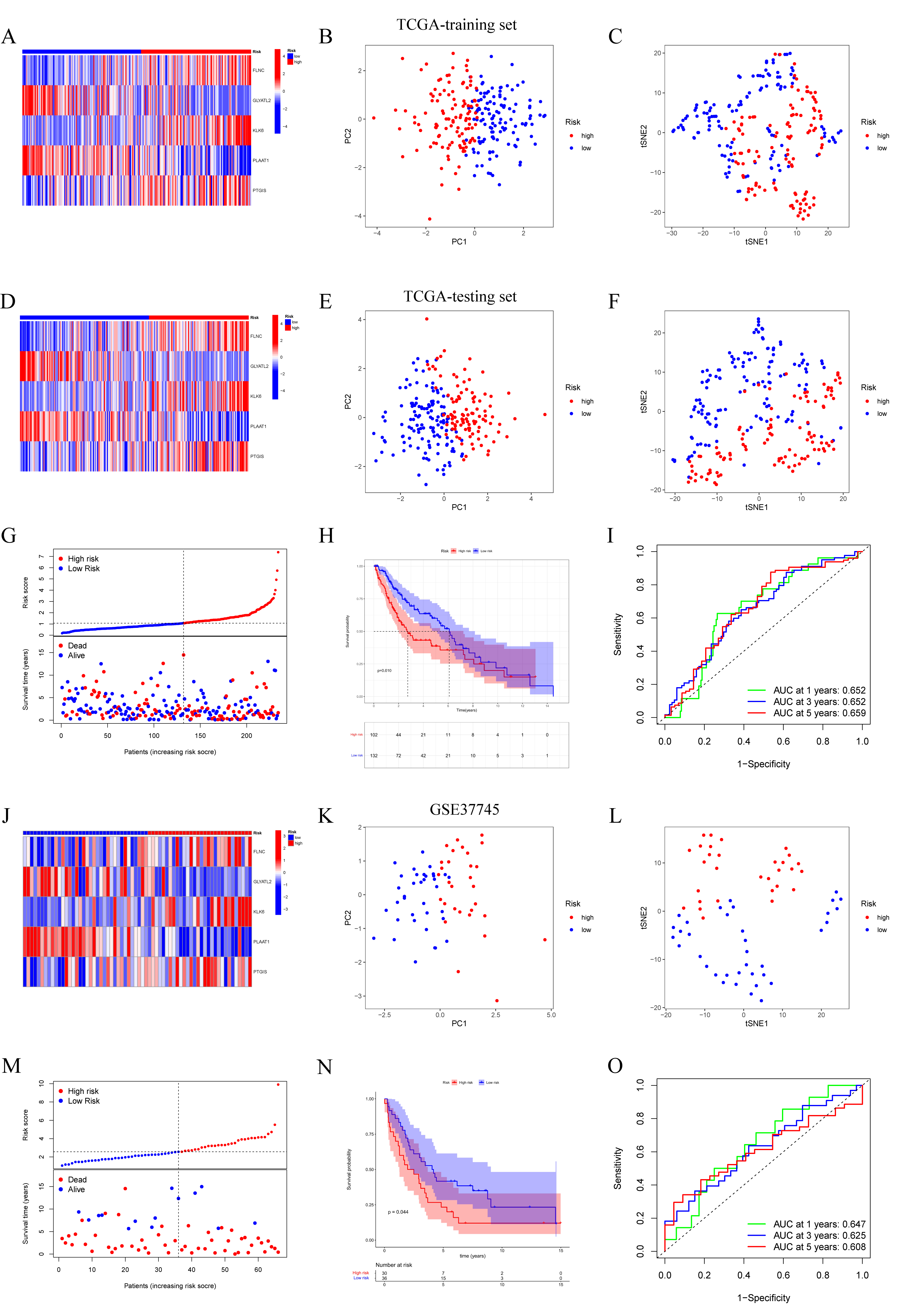

Supplement: Supplementary file 1 — Additional file 1: Fig. S1 Validation of prognostic signature for LUSC. [file 12672_2024_887_MOESM1_ESM.tif]

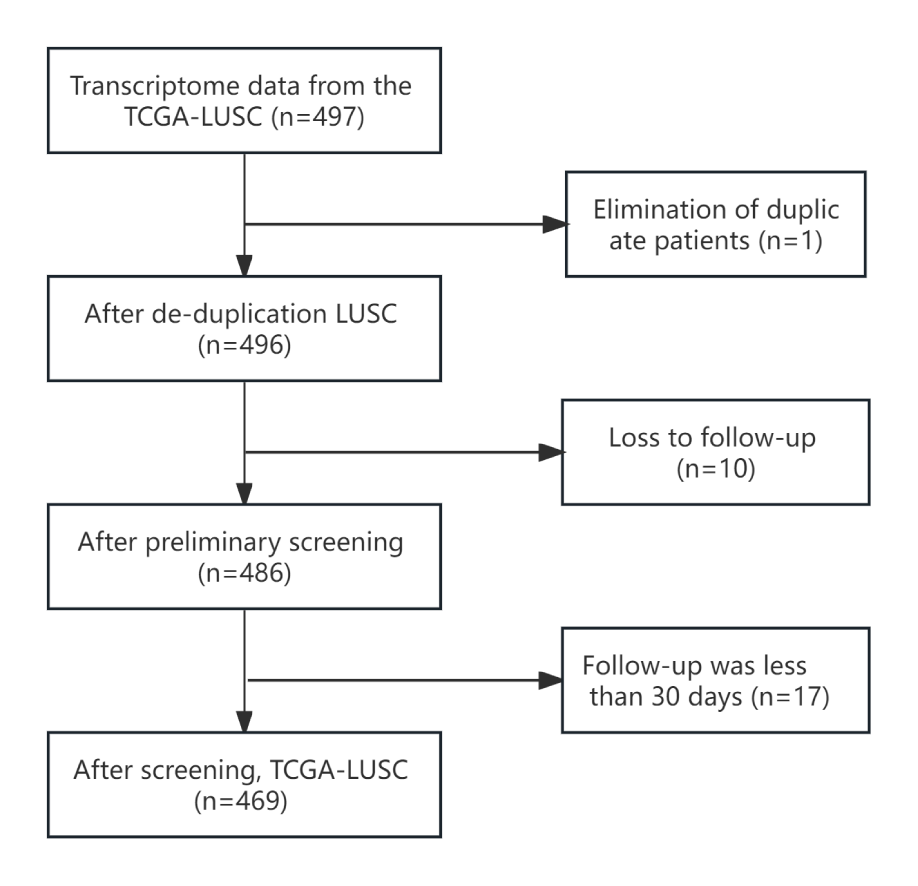

Supplement: Supplementary file 2 — Additional file 2: Fig. S2 LUSC patients screening process. [file 12672_2024_887_MOESM2_ESM.tif]
